# Cardiac Autonomic Neuropathy in Diabetes: A Predictor of Cardiometabolic Events

**DOI:** 10.3389/fnins.2018.00591

**Published:** 2018-08-27

**Authors:** Aaron I. Vinik, Carolina Casellini, Henri K. Parson, Sheri R. Colberg, Marie-Laure Nevoret

**Affiliations:** ^1^Division of Endocrinology and Metabolism, Strelitz Diabetes Center and Neuroendocrine Unit, Department of Medicine, Eastern Virginia Medical School, Norfolk, VA, United States; ^2^Department of Human Movement Sciences, Old Dominion University, Norfolk, VA, United States; ^3^VM BioPharma, Atlanta, GA, United States

**Keywords:** cardiac autonomic neuropathy, sympathetic, parasympathetic, dopamine deficiency, insulin resistance, cardiovascular event prediction, heart rate variability, diabetes

## Abstract

Autonomic nervous system (ANS) imbalance manifesting as cardiac autonomic neuropathy in the diabetic population is an important predictor of cardiovascular events. Symptoms and signs of ANS dysfunction, such as resting heart rate elevations, diminished blood pressure responses to standing, and altered time and frequency domain measures of heart rate variability in response to deep breathing, standing, and the Valsalva maneuver, should be elicited from all patients with diabetes and prediabetes. With the recognition of the presence of ANS imbalance or for its prevention, a rigorous regime should be implemented with lifestyle modification, physical activity, and cautious use of medications that lower blood glucose. Rather than intensifying diabetes control, a regimen tailored to the individual risk of autonomic imbalance should be implemented. New agents that may improve autonomic function, such as SGLT2 inhibitors, should be considered and the use of incretins monitored. One of the central mechanisms of dysfunction is disturbance of the hypothalamic cardiac clock, a consequence of dopamine deficiency that leads to sympathetic dominance, insulin resistance, and features of the metabolic syndrome. An improvement in ANS balance may be critical to reducing cardiovascular events, cardiac failure, and early mortality in the diabetic population.

## Introduction

As the United States population grows older and more obese, fully one third of people over the age of 65 will develop diabetes (Cowie et al., 2009; Boyle et al., 2010) and a host of comorbidities; and almost two thirds of these patients will experience cardiometabolic events. Unfortunately, the existence of comorbidities like metabolic syndrome, obesity, hyperlipidemia, and hypertension predict only about 24% of the risk of such events. Neuropathy (of all types) is one of the most prevailing complications of diabetes and a considerable source of morbidity and mortality; it includes DPN and AN (Vinik et al., 2013). AN, particularly when it involves cardiac function (CAN), independently predicts risk of cardiovascular death and myocardial infarction (MI) resulting in substantial morbidity and mortality (Pop-Busui et al., 2017).

It is likely that dysfunction of the ANS is an underdiagnosed cause of excessive morbidity and mortality in adults with diabetes. In fact, CAN results in cardiovascular (CV) dysfunction and is often accompanied by the progression to myocardial ischemia (which is often silent in diabetes), stroke, CAD, perioperative morbidity, and overall morbidity and mortality (Maser et al., 2003; Vinik and Ziegler, 2007; Pop-Busui et al., 2010). In combination with signs of DPN, the odds ratio increases to 4.55 for mortality and CVD (Vinik, 2010). This is a more robust indicator of CVD risk, exceeding lipoprotein profile, BP, as well as adenosine scans (Vinik et al., 2010). Thus, CAN assessments may be useful to predict cardiovascular risk.

## Cardiac Autonomic Neuropathy and Risk of Cardiovascular Events and Mortality

The prevalence of diabetes-related AN may be as high as 90% depending on the diagnostic methods and the cohort studied (Ziegler et al., 1992; Vinik et al., 2003). CAN prevalence increases substantially with diabetes duration in both T1DM (30% after 20 years) (Pop-Busui et al., 2009; Spallone et al., 2011; Martin et al., 2014) and T2DM (up to 60% after 15 years) (Low, 1996; Low et al., 2004; Spallone et al., 2011). In addition, CAN is present in some patients with prediabetes (Carnethon et al., 2006; Ziegler et al., 2008a, 2015b). CAN is strongly associated with risk of arrhythmias, major cardiovascular events, myocardial dysfunction, and cardiovascular mortality (Maser et al., 2003; Lykke et al., 2008; Ziegler et al., 2008b; Young et al., 2009; Pop-Busui et al., 2010, 2013). Consequently, early diagnosis of cardiac autonomic dysfunction is important. Several studies including ADVANCE, VADT, and ACCORD (Wackers et al., 2004; The Advance Collaborative Group, 2008; Duckworth et al., 2009; Pop-Busui, 2010; Pop-Busui et al., 2010; Zoungas et al., 2017) have shown that cardiac autonomic dysfunction may predict the risk of cardiovascular events and sudden death seen with intensification of glycemic control in subjects with T2DM. Significant escalations in cardiovascular events and all causes of mortality were independently correlated with resting baseline and average HR in a *post hoc* analysis of the ONTARGET/TRANSCEND studies, two large cohort studies of patients with stable, chronic CVD (Lonn et al., 2014). The influence of elevated resting HR and blunted HRV, two measures of ANS imbalance, on the development and progression of CVD, diabetes, and early mortality was assessed by Wulsin et al. (2015). In this Framingham Heart Study offspring cohort these measures, in addition to smoking, age, and gender, were shown to be significant predictors for developing CVD, DM, and early mortality within 12 years. Zafrir et al. (2016) also showed that resting tachycardia (HR > 100 beats/minute), chronotropic incompetence (inability of the heart to increase its rate commensurate with increased activity or demand), and reduced HR recovery after treadmill exercise were independently correlated with stroke, MI, and overall mortality in T2DM patients (hazard ratios of 1.97, 1.89, and 1.77, respectively), regardless of established coronary heart disease. In addition, the EURODIAB Prospective Cohort Study (Soedamah-Muthu et al., 2008) revealed that CAN was the most distinctive predictor for mortality in a large group of patients with T1DM, and increased mortality risk with deteriorating CAN was shown in a meta-analysis of several trials (Maser et al., 2003).

## Detection of Cardiac Autonomic Neuropathy

The most frequent clinical symptoms of CAN include dizziness, heart palpitations, lightheadedness, and fragility, all of which are consequences of dysregulation of the cardiovascular system secondary to malfunction of the sympathetic and parasympathetic nervous systems. Early autonomic dysfunction may exhibit no symptoms and only be detected by abnormal indices of HRV. Resting tachycardia (>100 bpm) and/or a fixed HR, as well as, orthostatic hypotension, intolerance to exercise, and syncope are present in patients with advanced CAN (The Consensus Committee of the American Autonomic Society and the American Academy of Neurology, 1996; Vinik and Ziegler, 2007; Pop-Busui, 2010; Spallone et al., 2011). In advanced stages diagnosis can be done clinically, but HRV tests may be necessary to detect early asymptomatic cardiac autonomic dysfunction. This can be easily performed in the office by utilizing an electrocardiogram recording during either 1–2 min of deep breathing, or as the subject begins to rise from a seated position, with calculation of HRV indices (Pop-Busui et al., 2017) (**Table 1**).

**Table 1:** Diagnostic tests for cardiovascular autonomic neuropathy (adapted from Brownlee et al., 2016).

**Resting heart rate**
Rate > 100 beats/min is abnormal
**Beat-to-beat heart rate variation^∗^**
With the patient at rest and supine (no overnight coffee or hypoglycemic episodes), breathing 6 breaths/min, heart rate monitored by ECG, an HRV of > 15 beats/min is normal and < 10 beats/min is abnormal, E/I ratio of R–R intervals > 1.17. All indices of HRV are age-dependent.^†^
**Heart rate response to standing^∗^**
During continuous ECG monitoring, the R–R interval is measured at beats 15 and 30 after standing. Normally, a tachycardia is followed by reflex bradycardia. The 30:15 ratio is normally > 1.03.
**Heart rate response to Valsalva maneuver^∗^**
The subject forcibly exhales into the mouthpiece of a manometer to 40 mm Hg for 15 s during ECG monitoring. Healthy subjects develop tachycardia and peripheral vasoconstriction during strain and an overshoot bradycardia and rise in blood pressure with release. The ratio of longest to shortest R–R interval should be > 1.2.
**Systolic blood pressure response to standing**
Systolic blood pressure is measured in the supine subject. The patient stands and the systolic blood pressure is measure after 2 min. Normal response is a fall of < 10 mm Hg, borderline is a fall of 10–29 mm Hg, and abnormal is a fall of > 30 mm Hg with symptoms.
**Diastolic blood pressure response to isometric exercise**
The subject squeezes a handgrip dynamometer to establish a maximum. Grip is then squeezed at 30% maximum for 5 min. The normal response for diastolic blood pressure is a rise of > 16 mm Hg in the other arm.
**Electrocardiographic QT/QTc intervals**
The QTc (corrected QT interval on ECG) should be < 440 ms.
**Neurovascular flow**
Non-invasive laser Doppler measures peripheral sympathetic responses to nociception.

^∗^These tests can be performed quickly (<15 min) in the practitioner’s office, with a central reference laboratory providing quality control and normative values, and are now readily available in most cardiology practices. ^†^Lowest normal value of expiration/inspiration (E/I) ratio by age is as follows: 20–24 year, 1.17; 25–29 year, 1.15; 30–34 year, 1.13; 35–39, 1.12; 40–44 year, 1.10; 45–49 year, 1.08; 50–54 year, 1.07; 55–59 year, 1.06; 60–64 year, 1.04; 65–69 year, 1.03; 70–75 year, 1.02. ECG, electrocardiogram; HRV, heart rate variation.

ADA recommendations regarding screening and diagnosis of CAN are as follows (Pop-Busui et al., 2017):

• “Symptoms and signs of AN should be assessed in patients with microvascular and neuropathic complications.
• In the presence of symptoms or signs of cardiovascular AN, tests excluding other comorbidities or drug effects/interactions that could mimic cardiovascular AN should be performed.
• Consider assessing symptoms and signs of cardiovascular AN in patients with hypoglycemia unawareness.”

The Toronto Consensus Panel, the European Society of Cardiology, the North American Society of Pacing and Electrophysiology, and the ADA Position Statement on Diabetic Neuropathy recommend the following regarding CAN assessments for clinical trials measuring a targeted intervention or for prognostication (Task Force of the European Society of Cardiology and the North American Society of Pacing and Electrophysiology, 1996; Bernardi et al., 2011; Pop-Busui et al., 2017):

• “Standardized CARTs: simple, sensitive, specific, and reproducible tests that assess changes in the R–R interval on electrocardiogram recordings in response to simple clinical maneuvers (deep breathing, Valsalva, and standing);
• Indices of HRV including sdNN and rMSSD;
• Resting HR and measurement of QTc interval on ECG recording”.

Cardiovascular autonomic reflex tests assess cardiovascular autonomic function through time-domain HR response to deep breathing, Valsalva maneuver and postural change, and by measuring the consequent changes in HR and BP. Although indirect autonomic measures, the following CARTs are considered the gold standard in autonomic testing: HR response to deep breathing (E:I ratio), standing (30:15 ratio) and Valsalva maneuver, and BP response to standing. The presence of one abnormal cardiovagal test result identifies possible or early CAN, to be confirmed over time. At least two abnormal cardiovagal results are required for a definite or confirmed diagnosis of CAN. The time-domain HR tests and the BP response to postural change have the reproducibility necessary for clinical trials.

### Time-Domain Measures of Heart Rate Patterns

Analysis of time-domain measures under resting conditions offers an accurate assessment of the sympathetic and parasympathetic regulation of the heart beat (the R–R interval on an electrocardiogram) documented at baseline conditions and during deep breathing, Valsalva, and standing from a sitting position maneuvers. The sdNN is an evaluation of both sympathetic and parasympathetic activity on HRV, and the rMSSD is a primary indicator of parasympathetic activity. Expiratory to inspiratory ratio (E/I ratio) measures HRV during deep breathing, which is parasympathetic predominant. Postural 30:15 ratio is evaluated at beats 15 and 30 after standing up and is regarded as reflective of sympathetic response and baroreflex function. Convertino (2014) demonstrated that cardiac parasympathetic withdrawal mediated by the carotid cardiac baroreflex is the principal trigger for tachycardia within milliseconds of a postural change, while sympathetic adrenergic control sustains tachycardia during extended periods of orthostasis. The Valsalva ratio is calculated by the longest R–R interval during the procedure to the shortest R–R interval throughout the duration or immediately following the maneuver. This reaction is facilitated by the interspersed activity of parasympathetic and sympathetic nerve fibers.

### Frequency-Domain Measures

Frequency-domain analysis can identify underlying periodicities in HR patterns. Rfa (also termed HF power) is calculated as the area under the HR spectral curve over a frequency range fixed on the fundamental Rfa (0.15–0.4 Hz), which is defined by the peak mode of the respiratory activity spectrum. LF is computed as the area under the HR spectral curve over the frequency range from 0.04 to 0.10 Hz (Fathizadeh et al., 2004; Vinik et al., 2011).

Despite its use in research for decades, the concept that LF and HF bands fully reflect separate influences of the sympathetic and parasympathetic branches has been recently under debate (Heathers and Goodwin, 2017), mainly due to their simultaneous action in the LF power (Valenza et al., 2018). Therefore, there has been increasing interest in using non-linear analyses of HRV, as these may be clinically more relevant by providing a better interpretation of the pathophysiological behavior of HRV under various conditions and by enhancing its prognostic value (de Godoy, 2016). Non-linear analysis methods do not assess the magnitude of variability but rather the quality, scaling, and correlation properties of the signals; these analyses allow a more subtle characterization of autonomic balance and have been shown to be more reliable markers of morbidity and mortality in patients with CVD. A number of studies have shown that abnormal non-linear HRV indices are associated with diabetes or an elevated risk of developing diabetes (Roy and Ghatak, 2013; Silva-E-Oliveira et al., 2017); and that non-linear HRV indices in diabetic populations may have diagnostic and prognostic potential for identifying asymptomatic CAN and cardiovascular events (Khandoker et al., 2009; de Godoy, 2016). The technical complexity of these analyses, however, has made interpretation and understanding of variability challenging for common clinical use. Further research is needed to demonstrate conclusively that these refinements in the analysis enhance the sensitivity for prediction of cardiovascular events (Sassi et al., 2015).

### Sudorimetry Measures

Sudomotor nerves are thin unmyelinated C-fibers, with largely cholinergic neurotransmission, where the ganglion neurotransmitter is acetylcholine, the primary parasympathetic nervous system neurotransmitter. However, epinephrine, norepinephrine, vasoactive intestinal peptide (VIP), atrial natriuretic peptide, calcitonin gene related polypeptide (CGRP), galanin, ATP, and substance P have been identified in periglandular nerves and thus may be contributing to the electrical response. The addition of sudomotor function assessments, combined with CART, may present a more precise and well-defined early diagnosis of ANS dysfunction.

Although quantitation of intraepidermal nerve fiber density on skin biopsies remains the gold standard and is the most recognized technique to diagnose small nerve fiber dysfunction, sudorimetry has the ability to produce diagnostic information on the evaluation of the small somatosensory nerves, detection and progression of disease, and responsiveness to therapeutic intervention. Sudorimetry technology has advanced rapidly as a non-invasive and precise tool to assess small fibers that can potentially be incorporated into clinical practice.

Current sudorimetry assessments can be performed using Sudoscan^TM^, which measures electrochemical skin conductance (ESC) of hands and feet. This technology is founded on the electrochemical theories of reverse iontophoresis and chronoamperometry to measure sudomotor function, which makes it an affordable, practical, and precise tool generating accurate profiles for routine clinical use and a viable research tool on the integrity of this complex system of control. This testing has assumed greater significance now that there are medications that can prevent the development or progression of ANS dysfunction. The American Association of Clinical Endocrinologists (AACE) endorses the use of current procedural technology (CPT) code 95923 for simplified sudomotor testing and the code 95943 for evaluation of cardiac autonomic function testing. AACE would urge that sudomotor function testing be authorized for all practitioners seeing patients with diabetes, including primary care, endocrinology, and podiatry. It is a non-invasive objective test, takes a mere 2 min, and it has been compared with other reference tests [including HRV indices, intraepidermal nerve fiber density, and quantitative sudomotor axon reflex testing (QSART)] and been demonstrated to be useful in the detection of small nerve fiber neuropathy in those with and without T2DM with a sensitivity of 77 to 87% and a specificity of 67 to 92% (AUC 0.77 to 0.88) (Casellini et al., 2013; Smith et al., 2014; Freedman et al., 2015; Selvarajah et al., 2015; Vinik et al., 2015, 2016).

## Medical Treatment of Cardiac Autonomic Neuropathy

Cardiac autonomic neuropathy therapies are typically focused on mitigating symptoms and should be directed to specified clinical manifestations. Exercise, volume repletion, low dose fludrocortisone and midodrine are among the most frequently used therapies. Recommendations for the treatment of CAN include the following (Pop-Busui et al., 2017):

• Early optimization of blood glucose regulation to avoid or delay the development of CAN in people with T1DM;
• Multifaceted approach to treat hyperglycemia and additional risk factors (e.g., dyslipidemia, hypertension) to prevent CAN in T2DM;
• Lifestyle modifications to improve CAN in patients with prediabetes.

Most recently there has been great interest in the action and effects of the sodium-glucose cotransporter-2 (SGLT2) inhibitors on reducing cardiovascular events. Empaglifozin is a highly selective inhibitor of the SGLT2 in the kidney. Glucose reduction occurs by decreasing renal glucose reabsorption and thereby increasing urinary glucose elimination in patients with diabetes, leading to significant reductions in glycated hemoglobin (HbA1c), weight loss, and reductions in BP without increases in HR (Liakos et al., 2014).

The EMPA-REG Outcome trial recruited 1,000 patients with T2DM, 700 of whom were enrolled and randomized to placebo or one of two different doses of empagliflozin (10 and 25 mg) daily in addition to standard care (Zinman et al., 2015). Empagliflozin was similar to other oral antihyperglycemic agents in HbA1c reduction (0.6–1%) and decreased both fasting and postprandial glucose, with a modest weight loss of ∼3 kg at 26 weeks vs. placebo, slightly greater weight loss at 52 weeks, modest BP reduction of 2–7 mmHg vs. placebo, and no intrinsic increased risk of hypoglycemia (Kishi, 2012). The initial report demonstrated a significant reduction in cardiovascular events (predominantly mortality and admission to hospital for heart failure) by 35% which fast tracked the drug to be approved for both diabetes and CVD. A subsequent report showed that the reduction in cardiovascular deaths were significant in Southeast Asia and Latin America, but not as much in America and Europe (Alzaid, 2017). Despite these different findings, the fall in BP without an increase in HR implies a reduction in sympathetic tone with its use.

Liraglutide, a GLP-1 receptor agonist, was also found to reduce CV events, but not as robustly as empagliflozin (Vinik et al., 2015). GLP-1 has widespread properties in the human body and targets receptors diffusely (Drucker, 2016). Liraglutide improves HbA1c and compared with other medication classes has similar or greater efficacy, even compared to basal insulin. Its use has been shown to lead to a modest improvement in BP but, in contrast to empagliflozin, with an increase in HR (Scirica et al., 2013; White et al., 2013; Green et al., 2015). The FDA recently approved the use of liraglutide for management of CVD in diabetes (Marso et al., 2016; Kumarathurai et al., 2017). The actions of liraglutide on HRV and daily variation of HR in newly diagnosed, overweight patients with T2DM and stable CAD have been investigated. Diurnal HR fluctuations and sympathovagal balance evaluated by rMSSD in NN intervals and HF and LF power were assessed. Liraglutide decreased sdNN in some subjects; decreased rMSSD; and increased mean, daytime, and nighttime HR compared to placebo. Liraglutide reduced HF power without any change in LF/HF ratio. Thus, in overweight patients with CAD and newly diagnosed T2DM, liraglutide increased HR and reduced HRV despite significant weight loss and improvement in metabolic parameters; the increase in nightly HR and decrease in parameters of parasympathetic activity (rMSSD and HF power) suggest that this medication may negatively affect sympathovagal balance (Kumarathurai et al., 2016). The authors hypothesize that the chronotropic effect of liraglutide, which may be mediated through the GLP-1 receptor on the sinoatrial node, cannot explain the worsening of HRV measures; instead, the impaired HRV may be due to a direct influence on sympathovagal balance, as reflected by the increase in night-time HR in conjunction with the significant decrease in sdNN and rMSSD suggesting an impairment of parasympathetic activity. The addition of a cholinergic agent to a GLP-1 analog might recapture the loss of cholinergic activity induced by a GLP-1 analog. This might even be a useful strategy to further enhance the cardiac protection afforded by the SGLT-2 inhibitors.

## Non-Medical Treatment of Cardiac Autonomic Neuropathy

A number of researchers have demonstrated that autonomic balance can be restored using simple lifestyle interventions, potentially reversing CAN. Motooka et al. (2006) showed that elderly women experienced improved HRV while walking a dog by enhancing parasympathetic function. Removing the dog resulted in reversal of this benefit with sympathetic overactivity (Motooka et al., 2006). There is strong evidence indicating that individuals with greater aerobic capacity exhibit enhanced HRV (Tulppo et al., 2003; Luque-Casado et al., 2013). Furthermore several studies have shown significant improvements in HRV measures after different training programs including cycling, walking, jogging and water aerobic exercise training in subjects with CAD (Laing et al., 2011), inspiratory muscle training in elderly individuals (Rodrigues et al., 2018), and high-intensity interval training in young, healthy individuals (de Sousa et al., 2018).

We have documented that falls and fractures in older diabetics were often the result of loss of organized variability, strength, and reaction times. Very simple strength and balance training can significantly reduce falls risk (Morrison et al., 2010). For patients with orthostatic hypotension, volume repletion with both fluids and salt is central to management, but physical activity and exercise are essential to prevent deconditioning, which is known to exacerbate orthostatic intolerance (Pop-Busui et al., 2017). The relationship between HRV and different psychiatric disorders, as well as stress and trauma, has also been extensively studied (Thayer et al., 2012). Subjects with depression and anxiety disorders exhibit abnormal HRV patterns compared with non-psychiatric controls (Servant et al., 2009). Reduced HRV characterizes emotional dysregulation, decreased psychological flexibility and defective social engagement, which in turn are linked to prefrontal cortex hypoactivity (Sgoifo et al., 2015). High occupational stress has also been associated with lowered HRV, specifically with reduced parasympathetic activation. There is limited evidence that use of biofeedback with relaxation and meditation approaches may result in increased HRV and parasympathetic activity (Servant et al., 2009). A more detailed review on this topic is beyond the scope of this article and the reader can refer to recent reviews on the subject.

## Prevention and Reversibility of Cardiac Autonomic Neuropathy

Prevention of CAN should be a primary focus of lifestyle and other clinical interventions. Intense glycemic control (The Diabetes Control and Complications Trial Research Group, 1995) utilizing a step-by-step progressive lowering of hyperglycemia, lipids, and BP, in addition to the use of antioxidants (Ziegler and Gries, 1997) and ACE inhibitors (Athyros et al., 1998), reduces the odds ratio for CAN (Gaede et al., 1999, 2008). In the EDIC study, patients with T1DM showed continuously favorable effects of past glucose control on microvascular complications in spite of the loss of glycemic separation (Diabetes Control and Complications Trial Research Group, 1993; Writing Team for the Diabetes Control and Complications Trial/Epidemiology of Diabetes Interventions and Complications Research Group, 2003). CAN progressed in both treatment groups during the EDIC follow-up, but the prevalence and incidence continued to be decreased in the previous intensive group compared to the standard group despite comparable levels of glycemic control. To diminish the development of CAN, intense glucose control of T1DM ought to be started as soon as possible (Pop-Busui et al., 2009). However, in patients with established CAN, glycemic control may need to be less stringent to avoid hypoglycemia and adverse drug effects (Inzucchi et al., 2015). The American Diabetes Association also recommends that individuals with CAN have a cardiac evaluation before starting or increasing physical activity for safety reasons (American Diabetes Association, 2017; Pop-Busui et al., 2017).

Pathogenesis-oriented interventions may promote some degree of reversal of established CAN (Vinik et al., 2011). Lifestyle interventions, increased physical activity, β-adrenergic blockers, aldose reductase inhibitors, ACE inhibitors, ARBs, and potent antioxidants such as α-lipoic acid have all been shown to restore autonomic balance. Enhanced glycemic control with a reduced HbA1c from 9.5 to 8.4 improved HRV in patients with T1DM with minimal autonomic abnormalities, but not in those with advanced autonomic irregularities (Burger et al., 1999). The Veterans Administration Cooperative Study showed no impact on the occurrence of CAN after 2 years of intense glycemic control in patients with T2DM (Azad et al., 1999); however, people with T2DM receiving rigorous multifactorial therapies that targeted hypertension, hyperglycemia, dyslipidemia, and microalbuminuria, along with secondary CVD preventative measures like aspirin use, experienced a 63% reduction in ANS dysfunction in the Steno-2 Study. Although glucose-lowering agents exerted the least benefit in comparison with antihypertensive treatments, lipid-lowering agents, aspirin, and vitamin-mineral supplements (Gaede et al., 1999), this favorable effect continued after ∼13 years of follow-up (Gaede et al., 2008). Early identification of CAN also may allow for the well-timed initiation of antioxidant alpha-lipoic acid therapies that slow or reverse advancement of CAN (Ziegler and Gries, 1997).

Certain medications hold promise for the prevention and reversal of CAN. Early therapeutic intervention with ACE inhibition or ARBs improved both CAN and left ventricular diastolic dysfunction after 1 year of treatment in patients with no symptoms and long-term diabetes. The combined therapies were slightly superior to monotherapies, auguring well for patients with established CAN (Didangelos et al., 2006). Treatment with fluvastatin improves cardiac sympathetic neuropathy in the diabetic rat heart in relation to attenuation of increased cardiac oxidative stress (Matsuki et al., 2010). Alternatively, selective inactivation of cyclooxygenase-2 (COX-2) guards against sympathetic denervation in experimental diabetes by decreasing intramyocardial oxidative stress and inflammation (Kellogg et al., 2009). Consequently, statins and COX-2 inactivation may assist in attenuating cardiac sympathetic dysfunction. In addition, it has been shown that early mortality is a consequence of beat-to-beat variability loss with MI, but can be reduced by 33% with the immediate administration of insulin (Malmberg et al., 1999). Successful pancreas transplantation showed improvements in epinephrine response and normalized hypoglycemia symptom awareness in patients with established diabetes (Burger et al., 1999) as well as evidence of a reversible metabolic component in patients with early CAN (Kendall et al., 1997).

Weight loss and weight-reducing surgeries may also potentially reduce CAN. ANS dysfunction and increased sympathetic activity have been directly correlated with obesity (Piestrzeniewicz et al., 2008; Straznicky et al., 2009; Lambert E. et al., 2010; Lambert G. W. et al., 2010). Moreover, weight reduction significantly improves HRV and reduces ANS imbalances (Karason et al., 1999; Maser et al., 2007; Nault et al., 2007; Perugini et al., 2010; Ravussin, 2010; Casellini et al., 2015, 2016; Ziegler et al., 2015a). To evaluate the ability to reverse autonomic imbalance, we examined sudomotor function and HRV measurements in obese patients undergoing bariatric surgery. Patients were assessed at baseline, 4, 12, and 24 weeks after vertical sleeve gastrectomy or Roux-en-Y gastric bypass. Seventy subjects completed at least 24-weeks of follow-up. Sudorimetry results of ESC of feet improved significantly trending toward normal in T2DM patients. HRV improved significantly, as did many other metabolic parameters. Improvements in feet ESC were shown to be independently associated with HbA1c, insulin, and HOMA2-IR levels at baseline, as well as HbA1c at 24 weeks. Additionally, improvement in basal HR had an independent association with HbA1C, insulin and HOMA2-IR levels. These positive results suggest that bariatric surgery can return both cardiac and sudomotor autonomic C-fiber dysfunction in those with diabetes to normal, possibly positively influencing morbidity and mortality (Casellini et al., 2016).

## Future Directions of Research to Prevent and Reverse Cardiac Autonomic Neuropathy

The host of targets that are potential candidates for reduction of cardiovascular risk have been addressed in the previous paragraphs. For years we were confronted with glycemic control as the only measure by the glucocentric majority and those who believed in the lipid hypothesis who have now carried this to the extreme of need for even lower LDL-C in high risk patients. The entry of SGLT2 inhibitors and the incretins shed new light on the challenge armed with new ammunition and also created an avenue of adventure for those interested in novel pathways. However the initial inroad into reduction of CV events was a discovery of the power of resetting a biologic clock and targeting the brain rather than other members of the dreadful dektet!

**FIGURE 1:**
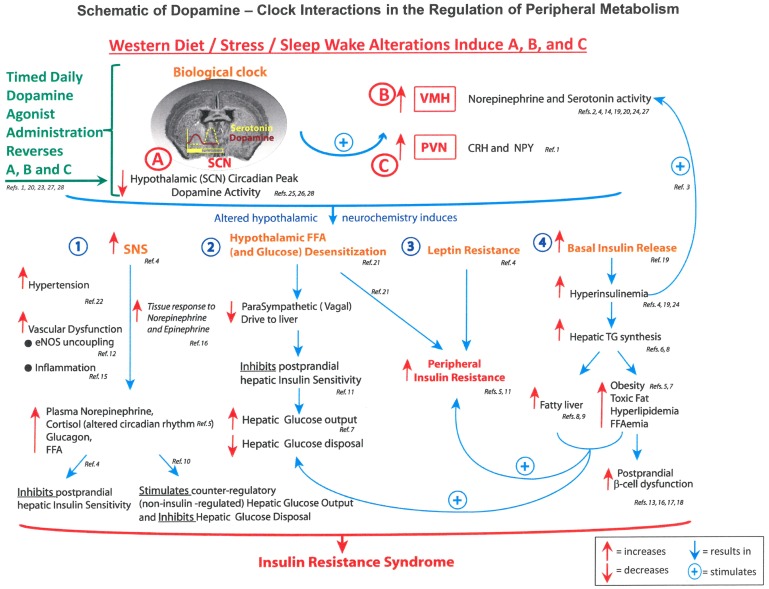
Schematic of dopamine – clock interactions in the regulation of fuel metabolism. Figure illustrates the hypothalamic clock with decreased suprachiasmic nuclear (SCN) early morning peak of dopamine activity and enhanced activity of the paraventricular nucleus (PVN), which increase autonomic tone and the paraventricular nucleus to raise levels of corticotrophin releasing hormone (CRH). The consequences include activation of the sympathetic nervous system (SNS) hypothalamic and glucose sensitization reducing parasympathetic (vagal drive) to the liver and resistance to both leptin and insulin. CRH, corticotrophin releasing hormone; eNOS, endothelial nitric oxide synthase; FFAs, free fatty acids; NPY, neuropeptide Y; PVN, paraventricular nucleus; SCN, suprachiasmatic nucleus; SNS, sympathetic nervous system; TGs, triglycerides; VMH, ventromedial hypothalamus (Raskin and Cincotta, 2016).

It has been established that there is a brain dopamine deficiency in obese diabetic patients present in the early hours of the morning (Cincotta et al., 1999). The working hypothesis is that in early morning, decreased dopaminergic tone in the hypothalamus unbridles sympathetic activation with all its consequences, as illustrated in **Figure 1**. Restoring the morning peak in dopaminergic activity by dopamine D2 receptor-mediated activities may, therefore, restore ANS balance.

It may also be possible to reset the biologic hypothalamic clock and ANS function using bromocriptine QR to restore morning dopaminergic activity. It sensitizes the body to insulin and reduces sympathetic tone thereby reducing HR (Raskin and Cincotta, 2016). Bromocriptine QR has also demonstrated a favorable effect on CV outcomes in clinical trials (**Figure 1** and **Table 2**) (Gaziano et al., 2012), suggesting a future direction for pathogenesis-oriented therapies (Vinik et al., 2011). Our current quest is to determine if any of the novel discoveries in cardiovascular outcome studies (CVOTs) are indeed working through rebalancing the ANS thereby creating a wonderful opportunity for taking a fork in the road.

**Table 2:** Impact of bromocriptine-QR on CV death-inclusive composite cardiovascular endpoint and individual components of the composite as well as the MACE endpoint.

	Bromocriptine-QR (*N* = 2054), n (%)^∗^	Placebo (*N* = 1016) n (%)^∗^	Hazard ratio (95% CI)
CV death-inclusive composite cardiovascular endpoint	39 (1.9)	33 (3.2)	0.61 (0.38 TO 0.97)
Myocardial infarction	7 (0.3)	9 (0.9)	0.41 (0.15 to 1.11)
Stroke	5 (0.2)	6 (0.6)	0.44 (0.13 to 1.43)
Hospitalization for angina	9 (0.4)	9 (0.9)	0.52 (0.21 to 1.30)
Hospitalization for heart failure	9 (0.4)	6 (0.6)	0.77 (0.27 to 2.16)
Coronary revascularization	11 (0.5)	8 (0.8)	0.72 (0.29 to 1.80)
CV death	4 (0.2)	2 (0.2)	0.48 (0.07 to 3.43)
Coronary revascularization following a primary endpoint (i.e., CABG after MI)	9 (0.4)	11 (1.1)	0.43 (0.18 to 1.03)
MACE composite-myocardial infarction, stroke, CV death	14 (0.7)	15 (1.5)	0.48 (0.23 to 1.00)

^∗^Percentage of events per total number per group; 2054 bromocriptine-QR, 1016 placebo. CI, confidence interval; CV, cardiovascular; CABG, coronary artery bypass graft; MACE, major cardiovascular adverse event; MI, myocardial infarction (Gaziano et al., 2012).

## Conclusion

An improvement in ANS balance may be critical to reducing cardiovascular events and early mortality. Symptoms and signs of autonomic dysfunction, including resting HR, BP responses to standing, and time and frequency measures of HRV in response to deep breathing, standing and Valsalva maneuver, should be elicited from all patients with diabetes to allow for early detection and intervention. With the recognition of the presence of ANS imbalance or for its prevention, a rigorous regime should be implemented with lifestyle modification, physical activity, and cautious use of medications that lower blood glucose. Rather than intensifying diabetes blood glucose management, a regimen tailored to the individual risk of ANS dysfunction should be constructed. The advent of new agents that may have the potential to improve ANS function, such as the SGLT2 inhibitors and the GLP-1 agonists, should be considered. However, it is not clear how these compounds work and what the mechanism of reduction of major adverse cardiovascular events is. An overlooked mechanism is a resetting of the biologic clock with correction of the dopamine deficiencies in the brainstem of obese people with diabetes, restoring the functioning of the ANS with its potential for significant reduction of cardiovascular events.

## Author Contributions

AV conceived of the presented idea and took the lead in writing the manuscript. CC and HP assisted in theory development, background research, and critical revisions to incorporate important intellectual content. SC participated in the manuscript development in the following ways: (a) substantial contributions to the conception or design of the work; or the acquisition, analysis, or interpretation of data for the work; (b) drafting the work or revising it critically for important intellectual content; (c) final approval of the version to be published; (d) agreement to be accountable for all aspects of the work in ensuring that questions related to the accuracy or integrity of any part of the work are appropriately investigated and resolved. M-LN critical review and manuscript editing.

## Conflict of Interest Statement

The authors declare that the research was conducted in the absence of any commercial or financial relationships that could be construed as a potential conflict of interest.

## Abbreviations

ACCORD: action to control cardiovascular risk in diabetes
ACE inhibitors: angiotensin-converting enzyme inhibitors
AN: autonomic neuropathy
ANS: autonomic nervous system
ARBs: angiotensin receptor blockers
BP: blood pressure
CAD: coronary artery disease
CAN: cardiac autonomic neuropathy
CARTs: cardiovascular autonomic reflex tests
COX-2: cyclooxygenase-2
CVD: cardiovascular disease
DCCT: diabetic control and complications trial
DM: diabetes mellitus
DN: diabetic neuropathy
DPN: diabetic peripheral neuropathy
EDIC: epidemiology of diabetes interventions and complications
GLP-1: glucagon-like peptide-1
HF: high frequency
HR: heart rate
HRV: heart rate variability
LF: low frequency
Lfa: low frequency area
MI: myocardial ischemia
Rfa: respiratory frequency area
rMSSD: root-mean-square of the difference of successive R–R interval
sdNN: standard deviation of all normal R–R intervals
T1DM: type 1 diabetes mellitus
T2DM: type 2 diabetes mellitus.
